# Reducing myocardial infarct size: challenges and future opportunities

**DOI:** 10.1136/heartjnl-2015-307855

**Published:** 2015-12-16

**Authors:** Heerajnarain Bulluck, Derek M Yellon, Derek J Hausenloy

**Affiliations:** 1 The Hatter Cardiovascular Institute, University College London, London, UK; 2 The National Institute of Health Research University College London Hospitals Biomedical Research Centre, London, UK; 3 National Heart Research Institute Singapore, National Heart Centre Singapore, Singapore, Singapore; 4 Cardiovascular and Metabolic Disorders Program, Duke-National University of Singapore, Singapore, Singapore

## Abstract

Despite prompt reperfusion by primary percutaneous coronary intervention (PPCI), the mortality and morbidity of patients presenting with an acute ST-segment elevation myocardial infarction (STEMI) remain significant with 9% death and 10% heart failure at 1 year. In these patients, one important neglected therapeutic target is ‘myocardial reperfusion injury’, a term given to the cardiomyocyte death and microvascular dysfunction which occurs on reperfusing ischaemic myocardium. A number of cardioprotective therapies (both mechanical and pharmacological), which are known to target myocardial reperfusion injury, have been shown to reduce myocardial infarct (MI) size in small proof-of-concept clinical studies—however, being able to demonstrate improved clinical outcomes has been elusive. In this article, we review the challenges facing clinical cardioprotection research, and highlight future therapies for reducing MI size and preventing heart failure in patients presenting with STEMI at risk of myocardial reperfusion injury.

## Introduction

For patients presenting with an acute ST-segment elevation myocardial infarction (STEMI), the most effective therapy for reducing myocardial infarct (MI) size, preserving LV systolic function and preventing the onset of heart failure, is timely reperfusion by primary percutaneous coronary intervention (PPCI). Although, myocardial reperfusion is a pre-requisite to salvaging viable myocardium, the process of restoring coronary blood flow can paradoxically induce myocardial injury and cardiomyocyte death, thereby mitigating the full benefits of reperfusion in terms of MI size reduction—a phenomenon which has been termed ‘myocardial reperfusion injury’.^1^ ^2^ The fact that a therapeutic intervention administered solely at the time of myocardial reperfusion can reduce MI size by up to half suggests that myocardial reperfusion injury may account for up to 50% of the final MI size.^1^ Although, the process of myocardial reperfusion has been optimised by advances in stent technology, new antiplatelet drugs (eg, prasugrel, ticagrelor and abciximab) and novel antithrombotic agents (eg, bivalirudin therapy), the latter being provided to maintain the rheology of blood flow, there is still no effective therapy for preventing myocardial reperfusion injury in PPCI patients. Following a STEMI, the size of the resultant myocardial infarct has been strongly linked with the development of adverse LV remodelling and heart failure,^3^ and subsequent prognosis following PPCI.^4^ Larose *et al*^5^ have shown that an MI size of ≥23% LV volume was associated with a HR of 6 for major adverse cardiac events.

In this article, we review the challenges facing clinical cardioprotection research, and highlight future opportunities for reducing MI size and improving clinical outcomes in patients presenting with STEMI at risk of myocardial reperfusion injury.

## Challenges facing clinical cardioprotection research

**Figure 1:**
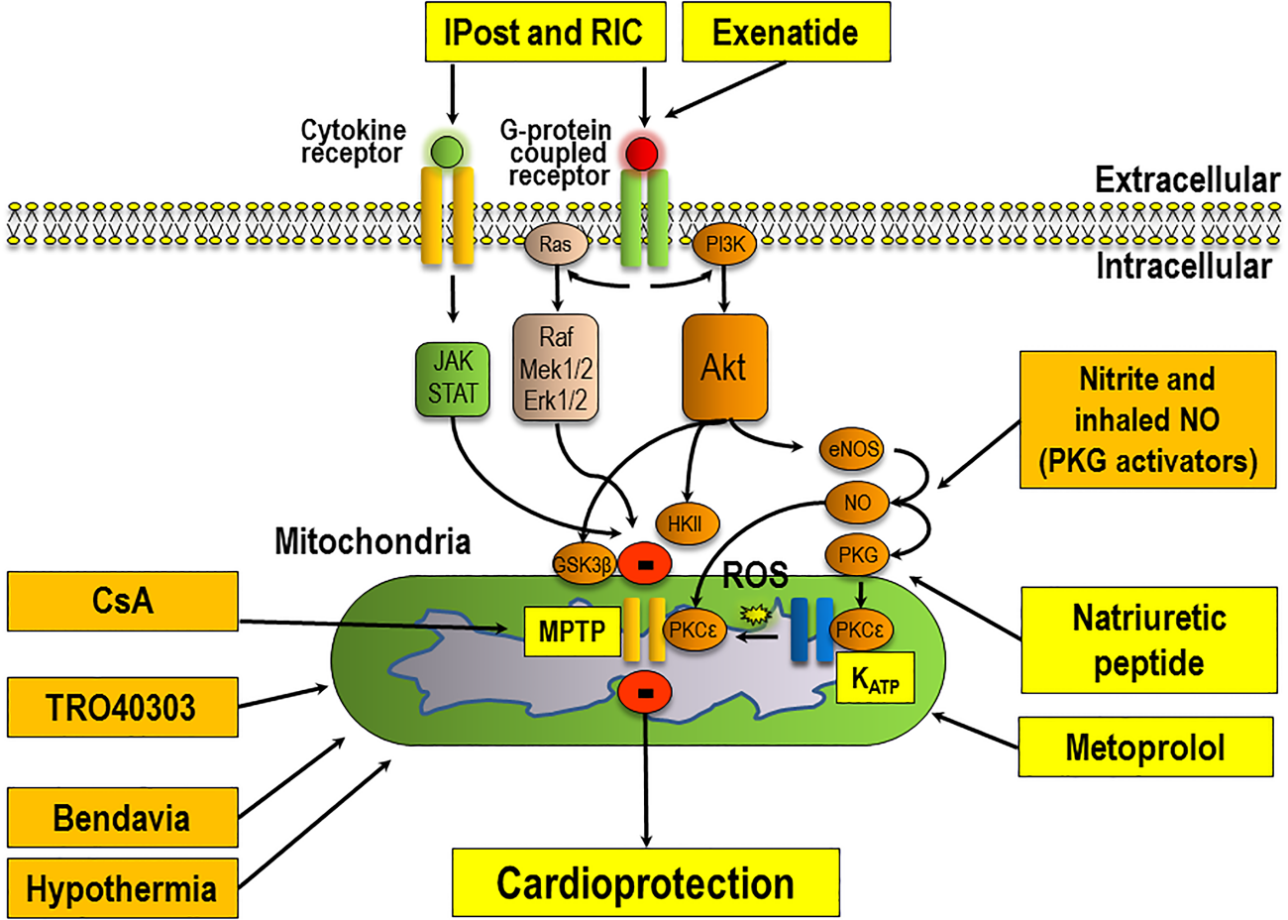
This scheme depicts the main prosurvival signalling pathways underlying ischaemic conditioning and the potential sites of actions for novel therapies which have recently been investigated in clinical studies to reduce myocardial infarct (MI) size in reperfused ST-segment elevation MI (STEMI) patients (please see tables 1–3, for details on the novel therapies and their potential sites of actions). The orange boxes indicate those therapies which have had mainly neutral effects on MI size and/or clinical outcomes (table 1) and the yellow boxes indicate those therapies which have the potential to improve clinical outcomes in reperfused patients presenting with STEMI (table 2). The signalling cascade underlying cardioprotection begins at the cardiomyocyte plasma membrane with the activation of G-protein coupled or cytokine receptors by autocoids such as adenosine, bradykinin or opioids (released in response to the ischaemic conditioning stimulus)—this results in the recruitment of signalling pathways such as the Reperfusion Injury Salvage Kinase (phosphatidylinositol 3-kinase-Akt (PI3K-Akt) and Mitogen-activated protein kinase kinase 1/2 -Extracellular signal-Regulated Kinase 1/2 (MEK1/2-Erk1/2)), Survivor Activator Factor Enhancement (SAFE), Janus kinase and Signal Transducer and Activator of Transcription (JAK-STAT) and the PKG pathways. These salvage pathways have been shown to activate downstream mediators such as endothelial Nitric Oxide Synthase (eNOS), Glycogen Synthase Kinase 3 Beta (GSK-3β), Hexokinase II (HKII), Protein Kinase C-epsilon (PKC-ε), the mitochondrial ATP-dependent potassium channel (KATP) which then mediate an inhibitory effect on mitochondrial permeability transition pore (MPTP) opening (adapted from Ref. 8).

There are a number of challenges facing clinical cardioprotection research. First, the translation of novel cardioprotective therapies into the clinical setting for patient benefit has been extremely difficult. Extensive literature exists on the complex array of prosurvival signalling pathways which underlie cardioprotection, and the readers are referred to a number of recent reviews^6^ ^7^—in figure 1, a simplified overview is provided which illustrates some of the major prosurvival pathways which mediate cardioprotection at the time of reperfusion. These have provided researchers with a number of molecular targets for pharmacological targeting of myocardial reperfusion injury. Over the past 30–40 years, a vast number of therapies with proven efficacy for preventing myocardial reperfusion injury and reducing MI size in experimental animal studies (eg, antioxidants, magnesium, calcium-channel blockers, anti-inflammatory agents, erythropoietin, atorvastatin, glucose-insulin-potassium therapy, adenosine) have produced disappointing results when investigated in the clinical setting as adjunctive therapy to reperfusion (reviewed in Ref. 9). More recently, a number of attempts to reduce MI size in patients presenting with STEMI have also failed to meet their primary end point of cardioprotection—these have included studies investigating therapeutic hypothermia, targeting mitochondrial function, and modulation of nitric oxide signalling as adjuncts to myocardial reperfusion (table 1 and figure 1).

**Table 1:** Recent attempts to reduce MI size in reperfused patients presenting with STEMI

Clinical study	Therapeutic intervention (patient population)	N	Main outcomes	Mechanism of cardioprotection potential reasons for neutral results
*Therapeutic hypothermia*				
Erlinge *et al*^10^ CHILL-MI	IV 600 to 2000 mL cold saline and endovascular cooling prior to PPCI for 1h to cool to 34.7°C	120	No effect on primary end point of MI size (CMR at 4 days) Delays reperfusion by 9 min	Experimental studies show that mild hypothermia, induced before reperfusion reduced MI size. The reasons for the neutral study are unclear but may relate to: 20%–27% of patients had TIMI flow of > 0 before PPCI Only 76% of patients had a temperature of <35°C at reperfusion Interestingly, post-hoc sub-group analysis revealed that patients presenting early (<4 h) with anterior STEMI had a smaller MI/AAR
Nichol *et al*^11^ VELOCITY	Peritoneal hypothermia to cool to 34.7°C	57	No effect on primary end point of MI size (CMR at 3–5 days) Delays reperfusion by 15 min Increase in stent thrombosis	The reasons for the neutral study may relate to: The study being underpowered. Out of 26 patients in the hypothermia arm, 6 had TIMI flow of >1 prior to PPCI and <50% achieved a temperature of <34.9°C at the time of PPCI
*Targeting mitochondrial function*				
Lincoff *et al*^12^ PROTECTION-AMI	IV delcasertib infusion for 2.5 h (LAD/RCA STEMI)	1010	No effect on primary end point of AUC CK-MB	Peptide inhibitor of delta-protein kinase C a major mediator of the mitochondrial apoptotic pathway which has been reported in animal studies to reduce MI size when administered prior to reperfusion.The reasons for the neutral study is unclear but may relate to: 30%–40% of patients had TIMI flow of >1 before PPCI. Intravenous delcasertib may take up to 30 min to reach steady state and therefore may not have been effective at reperfusion Lack of signs of toxicity raised the question of whether the dose used was sufficient
Atar *et al*^13^ MITOCARE	IV bolus of TRO40303 prior to angioplasty (All-comer STEMI, TIMI <1)	163	No effect on primary end point of 72 h AUC CK-MB/Troponin-T	This drug is an indirect inhibitor of the mitochondrial permeability transition pore which has been reported in animal studies to reduce MI size when administered prior to reperfusion.The reason for the neutral study is unclear but may relate to: Relatively small MI size compared with previous studies Groups were not well balanced (higher initial mean CK, less patients with TIMI 0 pre-PPCI and more patients with TIMI 0/1 post PPCI, older age by 4 years in the TR040303 group) Concerns that the dose used may not have been correct
Chakrabarti *et al*^14^ EMBRACE-STEMI	IV infusion (75 min) of Bendavia started 15 min before reperfusion (LAD STEMI, TIMI 0)	118	No effect on the primary end point of 72 h AUC CK-MB	A mitochondria-targeting peptide which has been reported in animal studies to reduce MI size when administered prior to reperfusion. The reason for the neutral study is unclear but may be because the study was underpowered. Full study results are awaited.
*Nitric oxide signalling*				
Siddiqi *et al*^15^ (NIAMI)	IV sodium nitrite (70 µmol) over 5 min prior to PPCI (All-comer STEMI, TIMI<1)	229	No effect on the primary end point of 72 h AUC CK-MB	The reason for the neutral study is unclear but may relate to the route of drug administration and the fact that >90% of patient had received GTN prior to reperfusion
Jones *et al*^16^ NITRITE	IC sodium nitrite (1.8 μmol) bolus prior to angioplasty (All-comer STEMI)	80	No effect on the primary end point of MI size (as % LV mass) (on 6–8 days CMR)	The reason for the neutral study is unclear but may relate to patient selection as post-hoc subgroup analysis revealed reduced MI size in patients with LAD STEMI
NOMI NCT01398384	Inhaled nitric oxide at 80 ppm for 4 h initiated prior to PPCI	248	No effect on the primary end point of MI size (as % of LV mass) on CMR (48–72 h)	The reason for the neutral study is unclear but may relate to patient selection (post-hoc subgroup analysis revealed reduced MI size in patients with LAD STEMI) and prior dosing with IC/IV GTN as patients who were GTN-naïve, there was a reduction in MI size

AUC, area under curve; CK-MB, creatine kinase myocardial band; CMR, cardiovascular MRI; GTN, glyceryl trinitrate; IC, intracoronary; IV, intravenous; LAD, left anterior descending artery; MI, myocardial infract; PPCI, primary percutaneous coronary intervention; RCA, right coronary artery; STEMI, ST-segment elevation myocardial infarction; TIMI, Thrombolysis in MI.

The reasons for this failure to translate cardioprotection into the clinical setting have been attributed to a number of factors including the use of inappropriate animal MI models and poorly designed clinical studies—a topic which has been discussed extensively in the literature and is highlighted in a following section.^9^ ^17^

A second challenge facing clinical cardioprotection research is that clinical outcomes of patients presenting with STEMI following PPCI continue to improve, making it increasingly difficult to demonstrate a reduction in MI size and improvement in clinical outcomes with a novel cardioprotective therapy. However, although mortality following STEMI is in decline, the number of patients surviving STEMI and going on to develop heart failure is increasing. There remains, therefore, an unmet need to discover novel therapeutic strategies capable of preventing myocardial reperfusion injury and reducing MI size, so as to preserve LV systolic function and prevent the onset of heart failure in patients presenting with reperfused STEMI. In the next section and in tables 2 and 3 we highlight a number of therapeutic strategies, which hold promise for reducing MI size and improving clinical outcomes in patients presenting with reperfused STEMI.

**Table 2:** Potential pharmacological strategies for reducing MI size and improving clinical outcomes in patients presenting with reperfused STEMI

Clinical study	Therapeutic intervention (patient population)	N	Main outcomes	Mechanism of cardioprotection and other comments
*Atrial natriuretic peptide*				
Kitakaze *et al*^18^	IV Carperitide (atrial natriuretic peptide analogue) 72 h infusion started prior to PPCI (All-comer STEMI)	569	15% reduction in MI size (72 h AUC total CK) 2.0% absolute increase in LVEF	Atrial natriuretic peptide targets prosurvival kinase pathways such as the cGMP and RISK pathways
*Ciclosporin A*				
Piot *et al*^19^ ^20^	IV Ciclosporin A (2.5 mg/kg Sandimmune) bolus 10 min prior to PPCI (LAD/RCA STEMI, TIMI 0)	58	44% reduction in MI size (72 h AUC total CK) 20% reduction in MI size (CMR subset) 28% reduction in MI size and smaller LVESV on CMR at 6 months	Ciclosporin A inhibits the opening of the mitochondrial permeability transition pore, a critical determinant of lethal myocardial reperfusion injury
Cung *et al*^21^ CIRCUS	IV Ciclosporin A (2.5 mg/kg Ciclomulsion) bolus 10 min prior to PPCI (LAD STEMI, TIMI 0)	791	No effect on the primary end point at 1 year of all-cause mortality, rehospitalisation for heart failure and adverse LV remodelling by echocardiography (59.0% Ciclosporin A vs 58.1% Control)	The reason for the neutral study is unclear but may relate to the Ciclomulsion preparation or failure of the drug to reach its molecular target. .
CYCLE NCT01650662	IV Ciclosporin-A (2.5 mg/kg Sandimmune) bolus 5 min prior to PPCI (LAD STEMI, TIMI 0/1)	410	*Ongoing study* Primary end point of ST-segment resolution ≥70% 1 h after PPCI	Recruitment complete October 2014—results awaited
CAPRI NCT02390674	IV Ciclosporin-A (2.5 mg/kg Sandimmune) bolus prior to PPCI (All-comer STEMI)	68	*Ongoing study* Primary end point MI size (3 month CMR)	
*Exenatide*				
Lonborg *et al*^22^ ^23^	IV infusion of Exenatide started 15 min prior to PPCI and continued for 6 h (All-comer STEMI, TIMI 0/1)	105	23% reduction in MI size (3 month CMR) Increase in myocardial salvage index (0.62–0.71) Short ischaemic times (≤132 minutes) associated with greater myocardial salvage	Exenatide, a GLP-1 analogue, targets prosurvival kinase pathways such as the RISK pathway
Woo *et al*^24^	S/C injection of Exenatide prior to PPCI (All-comer STEMI, TIMI 0)	58	52% reduction in MI size (1 month CMR) 27% reduction in MI size (72 h AUC CK-MB) 54% reduction in MI size (72 h AUC Trop-I)	
EMPRES NCT01938235	IV infusion of Exenatide for 24 h (All-comer STEMI, TIMI 0/1)	198	*Ongoing study* Primary end point of MI size at 3 months over area-at-risk at 72 h post randomisation (using CMR)	
*Metoprolol*				
Ibanez *et al*^25^ ^26^	IV Metoprolol (3×5 mg) in ambulance prior to PPCI (LAD STEMI)	270	20% reduction in MI size (7 day CMR) 3.7% absolute increase in LVEF (6 months CMR) 59% reduction in the incidence of poor LVEF (<35%) (6 months CMR) 65% reduction in need for ICD by 65% at 6 months 68% reduction in HHF at 2 years	The mechanism of cardioprotection is not clear.
Roovlink *et al* EARLY BAMI	IV Metoprolol (3×5 mg) in ambulance prior to PPCI (LAD STEMI)	408	*Ongoing study* Primary end point of MI size at 30 days on CMR	Ongoing study selecting patients with STEMI presenting within 12 h of onset of symptoms

AUC, area under curve; cGMP, cyclic guanosine monophosphate; CK, creatine kinase; CK-MB, creatine kinase myocardial band; CMR, cardiovascular magnetic resonance; GLP-1, glucagon-like peptide-1; HHF, hospitalisation for heart failure; ICD, implantable cardiac defibrillator; ICD, implantable cardiac defibrillator; IV, intravenous; LAD, left anterior descending artery; LVEF, left ventricular ejection fraction; LVESV, left ventricular end systolic volume; LVESV, left ventricular end-systolic volume; MI, myocardial infarct; PPCI, primary percutaneous coronary intervention; RCA, right coronary artery; RISK, reperfusion injury salvage kinase; STEMI, ST-segment elevation myocardial infarction; S/C, subcutaneous; TIA, transient ischaemic attack; TIMI, Thrombolysis in Myocardial infarction; Trop, troponin.

**Table 3:** Potential mechanical strategies for reducing MI size and improving clinical outcomes in patients presenting with reperfused STEMI

Clinical study	Therapeutic intervention/patient characteristics	N	Main outcomes	Comments
*Ischaemic postconditioning (major studies only)*				
Staat *et al*^27^	4×1 min angioplasty balloon inflations/deflations (All comer STEMI, TIMI 0)	30	36% reduction in MI size (72 h AUC Total CK)	First study to demonstrate reduction in MI size with IPost
Thibault *et al*^28^	4×1 min angioplasty balloon inflations/deflations (All comer STEMI, TIMI 0)	38	39% reduction in MI size (6 months SPECT) 7% absolute increase in LVEF at 12 months	First study to demonstrate long-term cardioprotection with IPost
Sorensson *et al*^29^	4×1-min angioplasty balloon inflations/deflations (All comer STEMI, TIMI 0)	76	No difference in MI size overall However, reduction in MI size in patients with large AAR	
Hahn *et al*^30^	4×1 min angioplasty balloon inflations/deflations (All comer STEMI, TIMI 0)	700	No difference in the primary end point of complete ST-segment resolution at 30 min post-PPCI	Largest neutral study with IPost. IPost performed at site of culprit lesion and MI size not measured.
Hofsten *et al* DANAMI-3^31^	4×0.5 min angioplasty balloon inflations/deflations (All comer STEMI, TIMI 0/1)	2000	*Ongoing study* Primary end point of all-cause mortality and heart failure at 2 years	Recruitment complete and follow-up in process Results due First study to investigate the effect of IPost on long-term clinical outcomes
*Remote ischaemic conditioning*				
Botker *et al*^32^	4×5 min arm cuff inflations/deflations in the ambulance prior to PPCI (All comer STEMI)	142	Increase in myocardial salvage index at 30 days No difference in MI size (SPECT or peak Trop-I)	First study to test effect of RIC in PPCI-treated patients presenting with STEMI. Reduced MI size in LAD STEMI.
Rentoukas *et al*^33^	4×4 min arm cuff inflations/deflations at the hospital prior to PPCI (All comer STEMI)	93	Better ST segment resolution and lower peak Trop-I. Synergistic effects with morphine	
Crimi *et al*^34^	3×5-min thigh cuff inflations/ deflations ***at onset*** of reperfusion by PPCI (LAD STEMI)	100	20% reduction in MI size (72 h AUC CK-MB)	First study to show benefit with RIC started at onset of reperfusion
White *et al*^35^ ERIC-STEMI	4×5 min arm cuff inflations/deflations at the hospital prior to PPCI (All comer STEMI)	197	27% reduction in MI size (acute CMR)	
Sloth *et al*^36^	4×5 min upper arm cuff inflations/deflations in the ambulance prior to PPCI (All comer STEMI)	251	51% reduction in composite end point of all-cause mortality, non-fatal MI, TIA or stroke, HHF at 3.8 years	First study to show an effect of RIC on long-term outcomes following PPCI (secondary end point)
Prunier *et al*^37^ RIPOST-MI	4×5 min arm cuff inflations/deflations at hospital prior to PPCI with or without IPost (All comer STEMI)	151	No difference in reduction in enzymatic MI size (CK-MB) with RIC vs RIC+IPost (31% vs 19%)	First study to test the effect of a combined therapeutic approach with RIC and IPost
Yellon *et al*^38^ ERIC-LYSIS	4×5 min arm cuff inflations/deflations at hospital prior to thrombolysis (All comer STEMI)	519	19% reduction in enzymatic MI size (CK-MB) 32% reduction in enzymatic MI size (Trop-T)	First study to test the effect of RIC in patients presenting with thrombolysed STEMI
Eitel *et al*^39^ 5 LIPSIA	3×5 min arm cuff inflations/deflations at hospital prior to PPCI with IPost (All comer STEMI)	696	Increased myocardial salvage index (CMR at 3 days) with RIC+IPost when compared with control (49 (IQR 30–72) vs 40 (IQR 16–68), p=0.02)	Largest study to test the effect of a combined therapeutic approach with RIC and IPost No beneficial effect with IPost alone
RIC-STEMI NCT02313961	3×5 min thigh cuff inflations/deflations prior to PPCI (All comer STEMI)	492	*Ongoing study* Primary end point of cardiac death and HHF at 12 months	
CONDI-2/ERIC-PPCI (NCT02342522)(NCT01857414)	4×5 min arm cuff inflations/deflations prior to PPCI (All comer STEMI)	4300	*Ongoing study* Primary end point of cardiac death and HHF at 12 months	Collaboration between UK, Denmark and Spain First study to investigate effect of RIC on long-term outcomes following PPCI

AAR, area-at-risk; AUC, area under curve; CK, creatine kinase; CK-MB, creatine kinase MB isoenzyme; CMR, cardiovascular magnetic resonance; HHF, hospitalisation for heart failure; IPost, Ischaemic postconditioning; LAD, left anterior descending artery; LVEF, left ventricular ejection fraction; MI, myocardial infarct; PPCI, primary percutaneous coronary intervention; RIC, remote ischaemic conditioning; SPECT, single-photon emission CT; STEMI, ST-segment elevation myocardial infarction; TIA, transient ischaemic attack; TIMI, Thrombolysis in Myocardial infarction; Trop, Troponin.

## Potential therapies for reducing MI size

### Ischaemic postconditioning: interrupting myocardial reperfusion to limit MI size

Interrupting myocardial reperfusion with several short-lived episodes of myocardial ischaemia has been demonstrated in experimental animal studies to prevent myocardial reperfusion injury and reduce MI size—a phenomenon which has been termed ‘ischaemic postconditioning’ (IPost).^40^ This therapeutic approach was rapidly translated into the clinical setting by Staat *et al*,^27^ who demonstrated that IPost (using four 1-min low-pressure inflations and deflations of the angioplasty balloon following direct stenting of the culprit lesion) led to a 36% reduction in MI size in PPCI-treated patients presenting with STEMI. Although a number of subsequent studies have reported similar beneficial findings with IPost, there have been a substantial number of neutral studies^29^ ^30^ (table 3). The reasons for this are unclear but it appears that most benefit is obtained in patients with anterior STEMI presenting with a completely occluded artery (TIMI 0).^29^ ^28^ Furthermore, the IPost protocol is best delivered following direct stenting and upstream of the stent rather than within it. Whether IPost can improve long-term clinical outcomes is not known, and is currently being investigated in the ongoing Danish Study of Optimal Acute Treatment of Patients with ST-Elevation Myocardial Infarction (DANAMI-3) trial.^31^

### Natriuretic peptide

Experimental animal studies have found that administering atrial natriuretic peptide (ANP) prior to myocardial reperfusion can reduce MI size through the activation of known prosurvival signalling pathways.^41^ Kitakaze *et al*^18^ translated this therapeutic approach in a large clinical study comprising 569 patients presenting with STEMI, in which administering Carperitide (an ANP analogue) at the time of PPCI was associated with a 14.7% reduction in enzymatic MI size (table 2). Further studies are required to confirm these findings and investigate whether this therapeutic approach can improve clinical outcomes in patients presenting with reperfused STEMI.

### Ciclosporin-A: targeting the mitochondrial permeability transition pore

The opening of the mitochondrial permeability transition pore (MPTP) in the first minutes of reperfusion is a critical mediator of reperfusion-induced cardiomyocyte death, and preventing its opening using pharmacological inhibitors of the MPTP such as ciclosporin-A (CsA) has been reported in experimental studies to limit MI size.^42^ The first study to translate this therapeutic approach into the clinical setting was by Piot *et al*^19^ who found in a small study of 58 patients presenting with STEMI that administering a single intravenous (IV) bolus of CsA prior to PPCI reduced MI size by approximately 40% as measured by creatine kinase (CK) (table 2). The recently completed CYCLosporinE A in Reperfused Acute Myocardial Infarction (CYCLE) study investigated the effect of MPTP inhibition using CsA (Sandimmune preparation) on ST-segment resolution, and the results of this study are eagerly awaited (table 2). Rather surprisingly, the large randomised Cyclosporine and Prognosis in Acute Myocardial Infarction Patients (CIRCUS) Phase III trial (comprising 791 patients presenting with acute anterior STEMI with data on the primary end point), which investigated the effect of CsA as an adjunct to PPCI on long-term clinical outcomes, failed to show any beneficial effect on the primary combined end point of all-cause mortality, hospitalisation for heart failure and adverse LV remodelling at 1 year^21^ (table 2). The reason for this neutral result is not clear—whether it relates to the Ciclomulsion preparation used in the study or failure of the drug to reach its target is not clear.^2^ Should MPTP inhibition ever be implemented as a therapeutic approach into the clinical setting, novel, more specific MPTP inhibitors will be needed, given the potential side effects and off-target effects of CsA.

### Exenatide: lizard saliva and cardioprotection

Exenatide (Byetta), a synthetic version of exendin-4 (a peptide isolated from the saliva of the Gila lizard), is a long-acting analogue of glucagon-like peptide-1 (GLP-1), a hormone which lowers blood glucose by stimulating insulin secretion.^43^ Interestingly, GLP-1 and its analogues such as exenatide have been reported in experimental animal studies to reduce MI size through the activation of prosurvival intracellular signalling pathways when administered prior to reperfusion.^44^ ^45^

Lonborg *et al*^23^ showed that initiating a 6 h infusion of exenatide prior to PPCI reduced MI size by 30% as measured by cardiovascular magnetic resonance (CMR) in patients presenting with STEMI, and most benefit was seen in those patients presenting <132 min from first medical contact.^22^ Woo *et al*^24^ also showed a significant reduction in MI size by CMR (26.4±11.6 g in the placebo arm vs 12.8±11.7 g in the exenatide arm: p<0.01) in 58 patients with subcutaneous exenatide (table 2). Whether this effect of exenatide portends to improved clinical outcomes in patients presenting with reperfused STEMI is not known and remains to be determined in a large randomised clinical trial.

### Remote ischaemic conditioning: transient limb ischaemia/reperfusion

Inducing brief non-lethal episodes of ischaemia and reperfusion in the arm or leg, using serial inflations and deflations (three to four 5 min cycles) of a standard blood pressure cuff placed on the upper arm or thigh, has been shown to protect the heart against acute ischaemia-reperfusion injury (IRI)—a phenomenon known as ‘remote ischaemic conditioning’ (RIC).^46–48^ It has been suggested that a blood-borne factor, of as yet unknown identity, is generated in response to the limb RIC stimulus, and this then transfers the cardioprotective stimulus from the limb to the heart where prosurvival intracellular signalling pathways are then activated and confer cardioprotection.^48^

A number of proof-of-concept clinical studies have demonstrated MI size reduction using limb RIC in patients presenting with STEMI treated by either PPCI^32–34^ ^37^ ^39^ ^49^ ^50^ and by thrombolysis^38^ (table 3). Whether this simple, non-invasive, low-cost, non-pharmacological intervention can improve long-term clinical outcomes in this patient group is currently being investigated in the Effect of RIC on Clinical Outcomes in STEMI Patients Undergoing PPCI and the Effect of Remote Ischaemic Conditioning on Clinical Outcomes in STEMI Patients Undergoing PPCI (CONDI2/ERIC-PPCI) trial. This European multicentre (Denmark, UK and Spain) randomised clinical trial of 4300 patients presenting with STEMI will determine whether upper limb RIC applied prior to PPCI can reduce the rate of cardiac death and heart failure hospitalisation at 1 year (ClinicalTrials.gov Identifiers: NCT02342522 and NCT01857414).

Repeated RIC post MI has attracted attention as a potential chronic cardioprotective therapy. Wei *et al*^51^ demonstrated in the rat heart that daily limb RIC for 28 days prevented adverse post-MI LV remodelling. The Chronic Remote Ischemic Conditioning to Modify Post-MI Remodeling (CRIC-RCT, NCT01817114) study and the Daily Remote Ischaemic Conditioning following Acute Myocardial Infarction (DREAM, NCT01664611) study are currently investigating this therapeutic strategy (daily RIC for 1 month) on post-MI LV remodelling following PPCI.

### Metoprolol: β-blockers and MI size reduction

Whether early β-blocker therapy is beneficial in patients presenting with reperfused STEMI is controversial and had not previously been investigated in the PPCI era. Using a porcine mode of acute myocardial IRI, Ibanez *et al*^52^ demonstrated that IV metoprolol administered prior to reperfusion resulted in a fivefold increase in myocardial salvage. The same researchers went on to apply this therapeutic strategy in the clinical setting by demonstrating that IV metoprolol administered in the ambulance prior to PPCI reduced MI size by 20% (assessed by CMR)^52^ and improved clinical outcomes in patients presenting with STEMI (as a secondary end point)^25^ ^26^ (table 2). Whether this pharmacological approach to reducing MI size can improve clinical outcomes will need to be tested in a large prospectively powered randomised controlled clinical trial.

### Combination reperfusion therapy: a novel therapeutic strategy

Prior attempts to reduce MI size in patients presenting with STEMI have relied on targeting one single component of myocardial reperfusion injury with a single agent. Whether, using combination reperfusion therapy can provide more effective cardioprotection against myocardial reperfusion injury remains to be investigated. In this regard, Alburquerque-Bejar *et al*^53^ have recently demonstrated an additive 26% reduction in MI size when combining RIC with insulin-like therapies (such as glucose-insulin-potassium and exenatide) in a porcine acute MI model. The Combination Therapy in Myocardial Infarction (COMBAT-MI) study (ClinicalTrials.gov Identifier: NCT02404376) will investigate the potential benefits of combined reperfusion therapy using RIC with exenatide on MI size reduction in patients presenting with STEMI treated by PPCI. Although an initial clinical study of 54 patients in patients presenting with reperfused STEMI failed to show an additive cardioprotective effect with RIC and IPost administered in combination,^37^ a recently published larger study of 696 patients found increased myocardial salvage in those patients administered RIC in combination with IPost when compared with controls (49 (IQR 30–72) vs 40 (IQR 16–68), p=0.02)^39^ (table 3).

## Optimising the clinical translation of cardioprotection

There are number of factors which need to be taken into consideration when designing clinical cardioprotection studies for reducing MI size in patients presenting with reperfused STEMI, as this may help facilitate the translation of novel cardioprotective therapies into the clinical setting for patient benefit.^9^

- *Patient selection*—where possible, consider selecting those patients presenting with STEMI who are most likely to benefit from the novel cardioprotective therapy—those presenting: early (<2–3 h as shown by Lonborg *et al*^22^ and Garcia-Dorado *et al*^54^); with a large area-at-risk (>30% of the LV volume)^29^ and an occluded artery prior to PPCI (TIMI <1) may be more likely to benefit from adjunctive therapy for reducing MI size. However, restricting patient selection has to be balanced with confining the testing of the novel cardioprotective therapy to a smaller selected group of patients.
- *The cardioprotective therapy*—consider only testing those novel therapies which have shown conclusive cardioprotection in a number of experimental small and large animal MI studies. In this regard, the NIH CESAR network in the USA offers the opportunity to test the cardioprotective efficacy of novel therapies in a multicentre small and large animal model setting akin to a multicentre clinical trial.^55^ Similarly, where possible it is important to demonstrate that the novel cardioprotective therapy is efficacious in several different clinical studies before proceeding to testing it in clinical outcomes studies.
- *Confounders of cardioprotection*—the presence of certain comorbidities (such as age, diabetes, pre-existing coronary artery disease, heart failure, hypertension, dyslipidaemia) and concomitant medication (nitrates, morphine, nicorandil, sulfonylureas, platelet inhibitors) may interfere with the efficacy of the novel cardioprotective therapy.^56^ Where possible this should be taken into consideration when designing experimental or clinical cardioprotection studies.
- *Timing of the cardioprotective therapy*—as myocardial reperfusion injury manifests in the first minutes of reperfusion, it is essential that the novel cardioprotective therapy is administered prior to myocardial reperfusion if it is to be effective. A number of neutral clinical studies were due to administering the cardioprotective therapy *after* reperfusion had already taken place.
- *Study end points*—consider selecting study end points which are most likely to be affected by the novel cardioprotective therapy—these include surrogate end points such as MI size (enzymatic, myocardial single-photon emission CT or CMR), myocardial salvage index (which is more sensitive than MI size reduction but requires the assessment of the AAR); microvascular obstruction (coronary no-reflow, index of microcirculatory resistance, or CMR), LV size and function and hard clinical end point such as heart failure hospitalisation, and cardiac death.

## Future perspectives

Clinical cardioprotection research remains a challenge—mortality rates following a PPCI-treated STEMI are in decline, which makes demonstrating a further reduction in MI size and improved clinical outcomes increasingly more difficult. However, an increasing number of patients are developing heart failure—as such, novel cardioprotective therapies which target myocardial reperfusion injury and reduce MI size provide the opportunity to preserve LV systolic function and prevent the onset of heart failure. The failure to translate a large number of infarct-limiting therapies into the clinical setting should not be taken as evidence that myocardial reperfusion injury does not exist in humans. Rather, it should provide the impetus to optimise the design of our experimental animal and clinical studies, and improve how we select which novel cardioprotective therapy to test in the clinical setting—this may hopefully facilitate the discovery of new effective therapies for reducing MI size and preventing heart failure in patients presenting with reperfused STEMI. Initial data suggest that using a combination of therapies to target myocardial reperfusion injury may be more beneficial than using a single therapy approach, and using this approach may result in improved clinical outcomes in this patient group.
